# Interactive abiotic and biotic stressor impacts on a stream‐dwelling amphibian

**DOI:** 10.1002/ece3.11371

**Published:** 2024-05-06

**Authors:** Oliver Coyle, Vance T. Vredenburg, Jonathon H. Stillman

**Affiliations:** ^1^ Department of Biology San Francisco State University San Francisco California USA; ^2^ Museum of Vertebrate Zoology University of California Berkeley Berkeley California USA; ^3^ Department of Integrative Biology University of California Berkeley Berkeley California USA

**Keywords:** behavior, *Dicamptodon tenebrosus*, ecological physiology, multistressor systems, stream amphibian

## Abstract

Organisms within freshwater and marine environments are subject to a diverse range of often co‐occurring abiotic and biotic stressors. Despite growing awareness of the complex multistress systems at play in aquatic ecosystems, many questions remain regarding how simultaneous stressors interact with one another and jointly impact aquatic species. We looked at multistress interactions in a protected stream ecosystem in Mendocino County, California. Specifically, we examined how diurnal temperature variation, turbidity, and predator cues altered the movement speed of larval Pacific giant salamanders (*Dicamptodon tenebrosus*). In a second experiment, we looked at how simulated low‐flow summer conditions impact the expression of heat‐shock proteins (HSPs) in the same species. Larvae moved almost one and a half times faster in the presence of chemical cues from trout and suspended sediment, and almost two times faster when both sediment and trout cues were present but were only marginally affected by temperature and visual cues from conspecifics. Interestingly, the order of stressor exposure also appeared to influence larval speed, where exposure to sediment and trout in earlier trials tended to lead to faster speeds in later trials. Additionally, larvae exposed to low‐flow conditions had more variable, but not statistically significantly higher, expression of HSPs. Our findings highlight the potential interactive effects of an abiotic stressor, sedimentation, and a biotic stressor, and predator chemical cues on an ecologically important trait: movement speed. Our findings also demonstrate the likely role of HSPs in larval salamander survival in challenging summer conditions. Taken together, these findings show that larval *D. tenebrosus* responds behaviorally to biotic and abiotic stressors and suggests a possible pathway for physiological tolerance of environmental stress. Consideration of multistress systems and their effects is important for understanding the full effects of co‐occurring stressors on aquatic organisms to guide appropriate conservation and management efforts based on ecologically relevant responses of organisms within an environment.

## Graphical abstract

Despite growing awareness of the complex multistress systems at play in aquatic ecosystems, many questions remain regarding how simultaneous stressors interact with one another and jointly impact aquatic species. In this study, we examined how diurnal temperature variation, turbidity, and predator cues altered the movement speed of larval Pacific giant salamanders (*Dicamptodon tenebrosus*), and how simulated low‐flow summer conditions impact the expression of heat‐shock proteins (HSPs) in the same species. In these larvae, we observed behavioral responses suggestive of interactive effects between abiotic and biotic stressors, and physiological responses that suggest a pathway for tolerance of environmental stress.

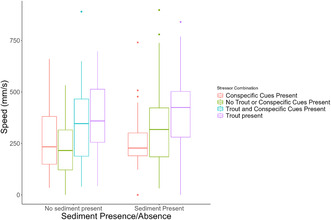

## 1 INTRODUCTION

Aquatic species in marine and freshwater environments face many stressors such as climate change (Stillman, 2019; IPCC, 2021), pollution (Huang et al., 2021), habitat modification (Huang et al., 2021; Reid & Dunne, 1984), and invasive species (Ruiz et al., 2000), each of which can have detrimental impacts on individuals and populations (Crain et al., 2008; Stillman, 2019). These stressors rarely occur in isolation, but rather occur simultaneously or in close succession with interactive effects (Gunderson et al., 2016; Todgham & Stillman, 2013). Such effects can be additive, antagonistic (exposure to one stressor decreases the severity of a second), or synergistic (the impact of multiple stressors is greater than the sum of the individual stressors) (Crain et al., 2008; Gunderson et al., 2017). The interaction that results is determined by contextual factors including the type, duration, and timing of stress exposure, prior conditions (Gunderson et al., 2017), and the species and environment in question (Harmon et al., 2009; Todgham et al., 2005).

Introduced stressors also occur alongside existing stressors such as inter‐ and intraspecific competition (Gunderson et al., 2017; Vafeiadou & Moens, 2021) and predation pressure (Hawlena & Schmitz, 2010; Pinya et al., 2016). These “native” stressors can augment the impact of external stressors and/or lower the threshold for which external stressors have negative impacts, although the extent of such impacts is again influenced by contextual factors such as species phylogeny and life history (Bernardo & Spotila, 2006; Harmon et al., 2009). Consequently, much work remains to better understand how biotic stressors in an environment interact with abiotic stressors to impact resident organisms.

According to the latest projections by the Intergovernmental Panel on Climate Change, average temperatures may increase by 1–4°C by 2060 and extreme weather events will increase in frequency and duration (IPCC, 2021), threatening numerous aquatic species (Hofmann & Todgham, 2010; Rosa & Seibel, 2008; Stillman & Somero, 2000) and altering aquatic ecosystems (IPCC, 2021; Moradkhani et al., 2010). Additionally, the combination of longer, more frequent droughts and consolidated precipitation events can increase sedimentation and turbidity in river systems (Allen et al., 2011; Welsh & Ollivier, 1998), further impacting aquatic ecosystems by decreasing microhabitat availability, inhibiting movement, lowering survival rates, decreasing abundance, and reducing reproductive success in fish (Kemp et al., 2011; Harvey et al., 2009), invertebrates (Rhett Jackson et al., 2007; Rosewarne et al., 2014), and amphibians (Corn & Bury, 1989; Welsh & Ollivier, 1998; Ashton et al., 2006; Kroll et al., 2008). These potentially detrimental sedimentation rises can also results from human activities (e.g., logging and construction) that can exacerbate the impacts of climatic change on sediment loads (Reid & Dunne, 1984; Stoddard & Hayes, 2005).

Considering these combined and likely worsening threats to aquatic ecosystems, it is vital to quantify how aquatic species and communities are impacted by multiple stressors co‐acting in their environment (Gunderson et al., 2016; Todgham & Stillman, 2013). To do so, it is important to target traits that are representative of individual fitness for species of interest that also influence their role in the ecosystem. Movement speed and escape behavior, for example, are crucial for individual survival and population stability (Cooper & Blumstein, 2015) and can be measured using minimally invasive methods through field‐based studies (Cooke et al., 2013; Madliger et al., 2018).

Similarly, physiological responses to environmental stressors can provide nonlethal indicators of the burden placed on aquatic organisms by current and future conditions (Burraco & Gomez‐Mestre, 2016; Gunderson et al., 2016; Todgham & Stillman, 2013). Components of the highly conserved cellular stress response (Kültz, 2005), for example, can help identify and measure the impact of environmental stressors on affected organisms and the capacity of those organisms to tolerate such conditions. Heat‐shock proteins (HSPs) are one component of the cellular stress response that are highly conserved across taxa (Kültz, 2005; Tomanek, 2008) and upregulated in the face of several environmental stressors such as temperature (Tomanek, 2008), dehydration (Luu et al., 2018), predation stress (Pauwels et al., 2005), and hypoxia (Kawabe & Yokoyama, 2011; Todgham et al., 2005).

Studies of the response of HSPs and movement speed to environmental stress can benefit from first studying the most sensitive species within an ecosystem, as they are likely to respond to stressors earlier or at lower levels than other species. These sentinel species can be helpful for monitoring of current conditions and predictions of broader ecosystem impacts (Rapport, 1992; Rapport et al., 1998). Amphibians, for example, are ideal species to study as indicators of ecosystem health (Davic & Welsh, 2004; Welsh & Hodgson, 2008) because they are exposed to terrestrial and aquatic stressors during different life stages (Salice et al., 2011; Wake & Vredenburg, 2008; Wilbur, 1980), and are particularly sensitive to many stressors (e.g., climate change (Cramp et al., 2021; Duarte et al., 2012), invasive species (Hawlena & Schmitz, 2010), and sedimentation (Kaufmann & Hughes, 2006)).

The giant Pacific salamander *Dicamptodon tenebrosus* (Baird and Girrard, 1852) is one of the most abundant amphibians in coastal and inland stream watersheds in the Pacific northwest (PNW) (Hawkins et al., 1983; Nussbaum & Clothier, 1973) and, as a top predator and key community member (Munshaw et al., 2013; Parker, 1994; Sánchez‐Hernández, 2020), helps shape community dynamics of invertebrate and vertebrate prey (Parker, 1994) and recycle nutrients (Munshaw et al., 2013). Specifically, *D. tenebrosus* larvae and paedomorphic adults are fully aquatic and thereby can come to dominate stream ecosystems at high trophic levels or influence the activity of other top stream predators. For example, *D. tenebrous* has a complex, direct relationship with salmonid fish such as steelhead trout (*Oncorhynchus mykiss*), characterized by mutual predation and dietary and microhabitat overlap (Lau, 1994; Parker, 1993; Rundio & Olson, 2003). Consequently, any changes to *D. tenebrosus* populations could have cascading consequences across streams and into estuaries in the PNW.

One watershed that harbors a high density of *D. tenebrosus* larvae is the Eel River watershed in Mendocino County in Northern California. The Eel River and its tributaries form one of the largest watersheds in California beginning at Bald Mountain in Mendocino County and emptying into the Pacific Ocean in Humboldt County (Brown & Ritter, 1971; Lisle, 1990; Yoshiyama & Moyle, 2010). The Eel River supports a wide range of aquatic species, including threatened chinook salmon, *Oncorhynchus tshawytscha*, and coho salmon, *Oncorhynchus kisutch*, and *O. mykiss* (Yoshiyama & Moyle, 2010). The Eel River also experiences high loads of suspended sediment (Brown & Ritter, 1971; Lisle, 1990) and will likely continue to be impacted by human activities that add to sediment production such as agriculture, road construction, and logging (Kaufmann & Hughes, 2006; Reid & Dunne, 1984; Sullivan, 2009). Consequently, understanding how resident organisms are impacted by sedimentation in concert with other stressors is key for watershed management and species conservation.

Given the key trophic position and projected sensitivity of *D. tenebrosus* larvae to environmental stressors, we looked at the isolated and joint impacts of abiotic (i.e., temperature, sedimentation) and biotic stressors (chemical and visual predator cues) on movement speed and escape behavior in *D. tenebrosus* larvae. Additionally, to investigate physiological response to multistress systems, we assessed accumulation of HSPs in *D. tenebrosus* larvae exposed to temperature challenges that accompany low‐flow conditions during the warmest summer months in coastal California watersheds.

## 2 METHODS

### 2.1 Animal collection and measurement

Giant Pacific salamander, *Dicamptodon tenebrosus*, larvae (*n* = 56) were captured in the Fox Creek tributary of the south fork Eel River at the Angelo Coast Range Reserve in Mendocino County, California, during June 2022. Captures occurred along the lower stem of Fox Creek between 39.740424 and −123.632784 and 39.740824 and −123.629910. Larvae are fully aquatic unlike terrestrial adults and distinguished from neotenic adults by size (larvae usually metamorphosize or cross over into neoteny between 92 and 166 mm on length) (Nussbaum & Clothier, 1973). All individuals were caught using dip netting (8‐inch Top Fin® Fish Net) and hand capture and were placed in in situ enclosures in the stream that allowed for natural water flow and light conditions. These enclosures were made by cutting the sides off 5‐gallon plastic buckets and covering the opening with nylon mesh too fine to allow the salamander to escape. Mesh lids were placed over top to prevent salamanders from climbing out of the buckets. No larvae mortalities were observed during the trials, and all larvae were released at the site of original capture in Fox Creek at the Angelo Coast Range Reserve at the conclusion of trials. All captures were performed using an approved San Francisco State University IUCAC protocol (A20‐05‐SFSU, Vance Vredenburg) and California Department of Fish and Wildlife Scientific Collecting Permit (S‐190980001‐19111‐001, Vance Vredenburg) and with approval from the Angelo Coast Range Reserve.

After capture, all individuals were measured to the nearest mm and 0.5 g using a ruler, calipers, and spring scale. The snout‐ventral length in mm (SVL), tail length in mm (TL), total length in mm, and weight in grams of each specimen were recorded. Additionally, any injuries, included missing tails and missing limbs, were recorded.

### 2.2 Escape behavior assay

Escape behavior was monitored during exposure of larvae to environmental stressor conditions in an assay enclosure. To start the escape behavior assay, a larva was removed from its in situ enclosure and placed in a larger 1.22 by 0.46 m rectangular, plastic container filled with stream water and three rocks collected from the stream as cover objects. Water in the assay enclosure was still and replaced with fresh water from the stream following each treatment, at approximately 10‐ to 15‐min intervals. Rocks were evenly spaced along one of the longer sides of the enclosure. During all trials, the water temperature in the assay enclosure was recorded using a thermocouple.

In the first set of escape behavior assays, larvae (*n* = 25) underwent two trials (one morning and one afternoon) per day for 4 days in (1) control conditions (water from the stream with no added chemical cues, sediment, or visual predator cues), (2) in the presence of chemical cues collected from *Oncorhynchus mykiss*, (3) in the presence of visual cues from a conspecific, or (4) in the presence of high suspended sediment. All four of these conditions were tested twice, once in the morning and once in the afternoon over the course of 4 days such that each salamander underwent a trial during both times. Water containing chemical cues from steelhead trout was collected from permanent tanks holding adult trout at the Fangue Lab at the University of California Davis, aliquoted into 100 mL bottles, frozen (−20°C), and stored (−20°C) until use to ensure the predator cue remains chemically active (Brown & Smith, 1998). Chemical cue‐infused water (100 mL/50 L of water) was introduced and mixed into the trial enclosure prior to trout cue trials. To provide visual cues, a third‐year conspecific larva was placed in a glass container inside the larger enclosure during escape behavior trials. This individual was one of three individuals who had already completed their trial on the given day and time. Natural clay was used to add suspended sediment (2000 mg/L) to the enclosure. The water in the enclosure was replaced by emptying the enclosure and refilling it from the stream using a 5‐gallon bucket between treatments to mitigate unwanted interactive effects of different treatments. There was at least a 5‐h gap between morning and afternoon trials to maximize rest time between trials with available daylight.

A new group of larvae (*n* = 24) was captured for a second behavioral assay. This assay followed the same 4‐day morning and afternoon format as the first assay, but combined the environmental stressors from the first assay. Larvae underwent trials in (1) control conditions (as described above), (2) in the presence of sediment and visual cues from a conspecific, (3) in the presence of chemical cues from steelhead trout and visual cues from a conspecific, (4) or the presence of sediment and chemical cues from steelhead trout.

Analysis of escape behavior (e.g., types of movement) and movement speed was performed from video recordings of the behavioral assays. All trials were video recorded with an iPhone 13 held over the top of the larger enclosure. Videos from the escape behavior assays were analyzed in Fiji (ImageJ) using the FFmpeg package. For each video, the scale of pixels to mm was set using the known length of the salamander in the video (Arismendi et al., 2021). After the scale was set, the distance and duration traveled were calculated to determine the speed of the salamander following each disturbance. The number of times the salamander went to cover was also recorded.

### 2.3 Statistical analysis of escape behavior

All statistical analyses were performed in R studio (2022.07.2, Build 576) using the tidyverse (Wickham et al., 2019), lme4 (Bates et al., 2015), and lmertest (Kuznetsova et al., 2017) packages (RStudio Team, 2021). Max speed data were normally distributed. The speed of salamanders was compared among treatments using ANOVA and Tukey HSD tests, and a GLMM was used to compare the impact of treatment on max speed. Max speed was used as the response variable, the treatment as a fixed variable, and water temperature, size, and the injury status of the individuals as random effects. The relation between treatment and amount of time spent under cover was analyzed using ANOVA and a Tukey HSD test. Additionally, Chi‐squared tests were used to comparing the predicted escape success of salamanders against published strike speeds of known predators including trout (Webb & Zhang, 2011), garter snakes (Alfaro, 2002), and conspecifics (Kleinteich et al., 2014; Reilly & Lauder, 1992). Percent change between speeds for individual salamanders under different treatments compared to controls were log‐transformed and analyzed using GLM, ANOVA, and Tukey HSD. For analysis purposes, measured temperatures during the escape behavior trials were grouped into low—less than 11.3°C, medium—between 11.3 and 15.5°C, and high—greater than 15.5°C based on in‐stream measured temperatures from November 2021 to October 2022 with a HOBO TidBit 400′ data logger (Onset Co., Bourne, MA).

### 2.4 Low‐flow simulation trial

A new group of larvae (*n* = 30) were captured for a simulation of low‐flow conditions. All larvae were again placed in the in situ enclosures used during the escape behavior trials. Half of the enclosures (*n* = 30, *n* = 15 per treatment) were placed in the stream to allow for natural stream flow conditions, and the other half were placed on the stream bank filled with six inches of water so the larvae were submersed but not experiencing natural stream flow. This second group of enclosures mimicked the small pools where larvae become trapped during low‐flow conditions in summer, a time of high mortality for this age class (Nussbaum & Clothier, 1973). Specifically, those trapped in the “low flow” enclosures experienced higher temperatures (recorded by thermocouple) than those in the stream (temperature recorded by HOBO logger). Larvae in the low‐flow condition were seen spreading their gills and flushing them with blood, a behavior likely used to increase surface area and gas exchange. This behavior was not seen in individuals in the stream. The two groups remained in their respective enclosures for 48 h, after which tail clips were collected from larvae in both exposed and control enclosures. Tail clips have been shown to be a minimally damaging manner of collecting tissue from salamanders with regenerative capabilities, such as *D. tenebrosus* (Othman et al., 2020; Segev et al., 2015). Tail clips were stored in cryogenic vials, frozen in liquid nitrogen vapor, and transported back to the Stillman Lab for storage at −80°C for analysis of heat‐shock protein 70 (HSP70) levels.

### 2.5 HSP analysis

Frozen tail clips were thawed and homogenized in a 10:1 dilution in lysis buffer (32 mM tris‐glycine pH 6.8 with 2% SDS and 1 μM PMSF) using a rotor‐stator homogenizer (Power‐Gen; Fisher Scientific), boiled at 100°C for 5–10 min, and then centrifuged at 16,000 *g* for 15 min. The supernatant was then aliquoted and frozen at −20°C.

Supernatant (60 μL) was mixed with Laemmeli blue dye (4:1) prior to loading onto a precast SDS gel (Mini‐PROTEAN® TGX™ Precast Gels; Biorad, Hercules, CA, USA). Protein standards (Precision Plus Protein™ Kaleidoscope™ Prestained Protein Standards, #1610375; Biorad, Hercules, CA, USA) and purified HSP (rat HSP70 HEK293 Overexpression Lysate; Sinobiological, Wayne, PA, USA) were added as controls. Proteins were separated in 1× tris‐glycine/SDS buffer at constant of 200 V until maximal separation in the standard ladder was achieved. Proteins were transferred from the gel to a Millipore Immobilon nitrocellulose membrane at 100 V at room temperature for 1 h using a tris‐glycine buffer (25 mM Tris, 192 mM glycine, and 0.1% (w/v) SDS, pH 8.3) with 20% methanol.

After transfer, the membrane was blocked in Intercept PBS blocking buffer (#927‐70001; Li‐Cor Biosciences, Lincoln, Nebraska, USA) for at least 1 h. The membrane was then placed in 1:5000 diluted primary antibody (mouse monoclonal [N27F3‐4] to HSP70, Abcam, Cambridge, UK) for 1–2 h and then washed 3× for 5 min in 1% phosphate‐buffered saline (PBS) solution with 0.1% Tween‐20 (1% PBST). Subsequently, the membrane was placed in the 1:15,000 diluted secondary antibody (Goat Anti‐Mouse IgG H&L (Alexa Fluor® 680), Abcam) for 30 min, washed first 3× for 5 min with 1% PBST, and then 2× for 5 min with 1% PBS. All blocking and binding occurred at room temperature using a shaking platform.

Membranes were imaged using an Odyssey CLx scanner (LI‐Cor biosciences, Lincoln, Nebraska, USA) using the 700 nm channel and quantified using the built‐in software of the imaging device. Recorded signals were then converted to μg/mL concentration using the known concentration of positive controls and volume of samples ran in gels.

## 3 RESULTS

### 3.1 Escape behavior

**FIGURE 1:**
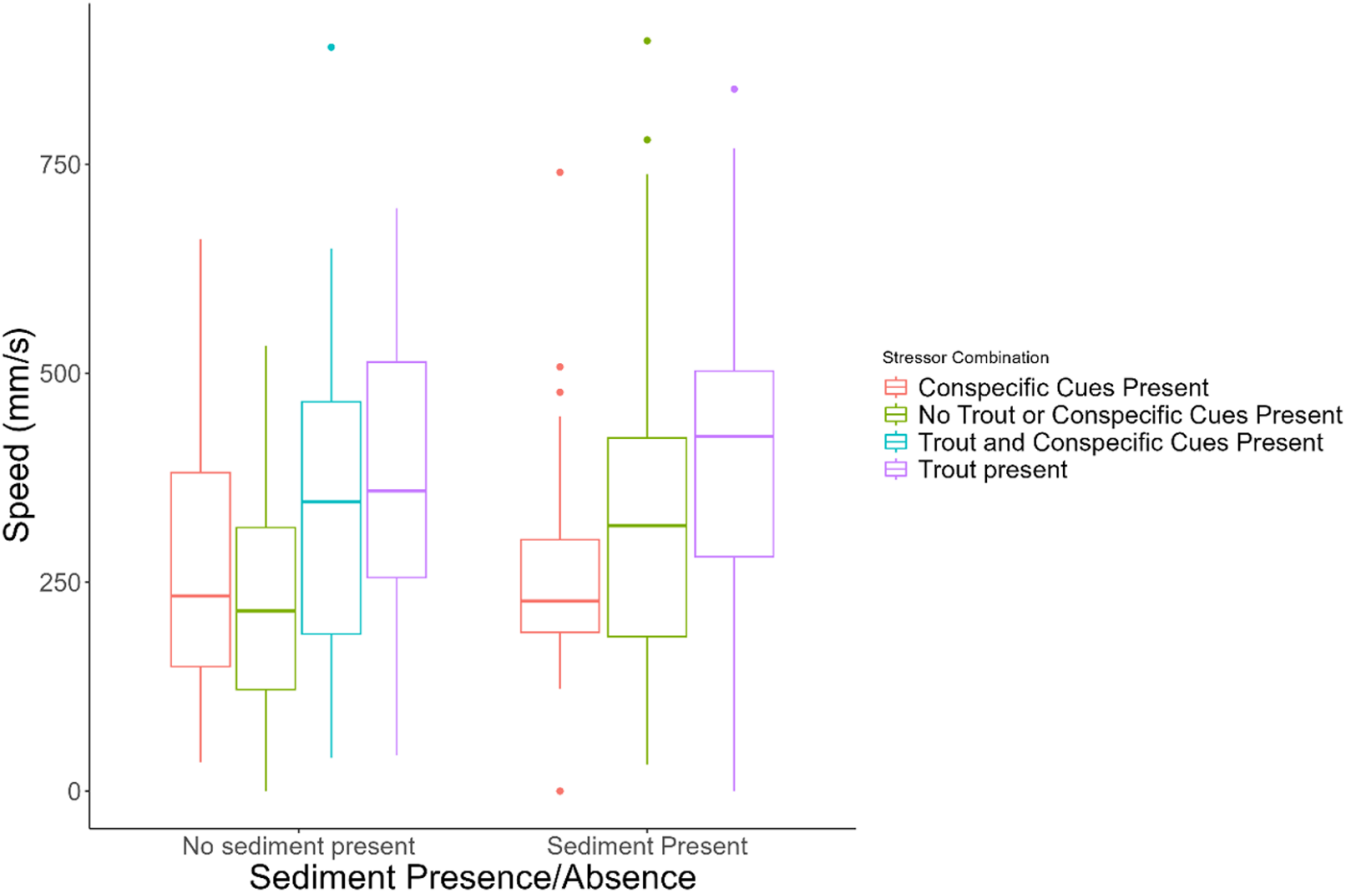
The effects of sediment turbidity, conspecific visual cues, and predator chemical cues on movement speed of *Dicamptodon tenebrosus* larvae.

**FIGURE 2:**
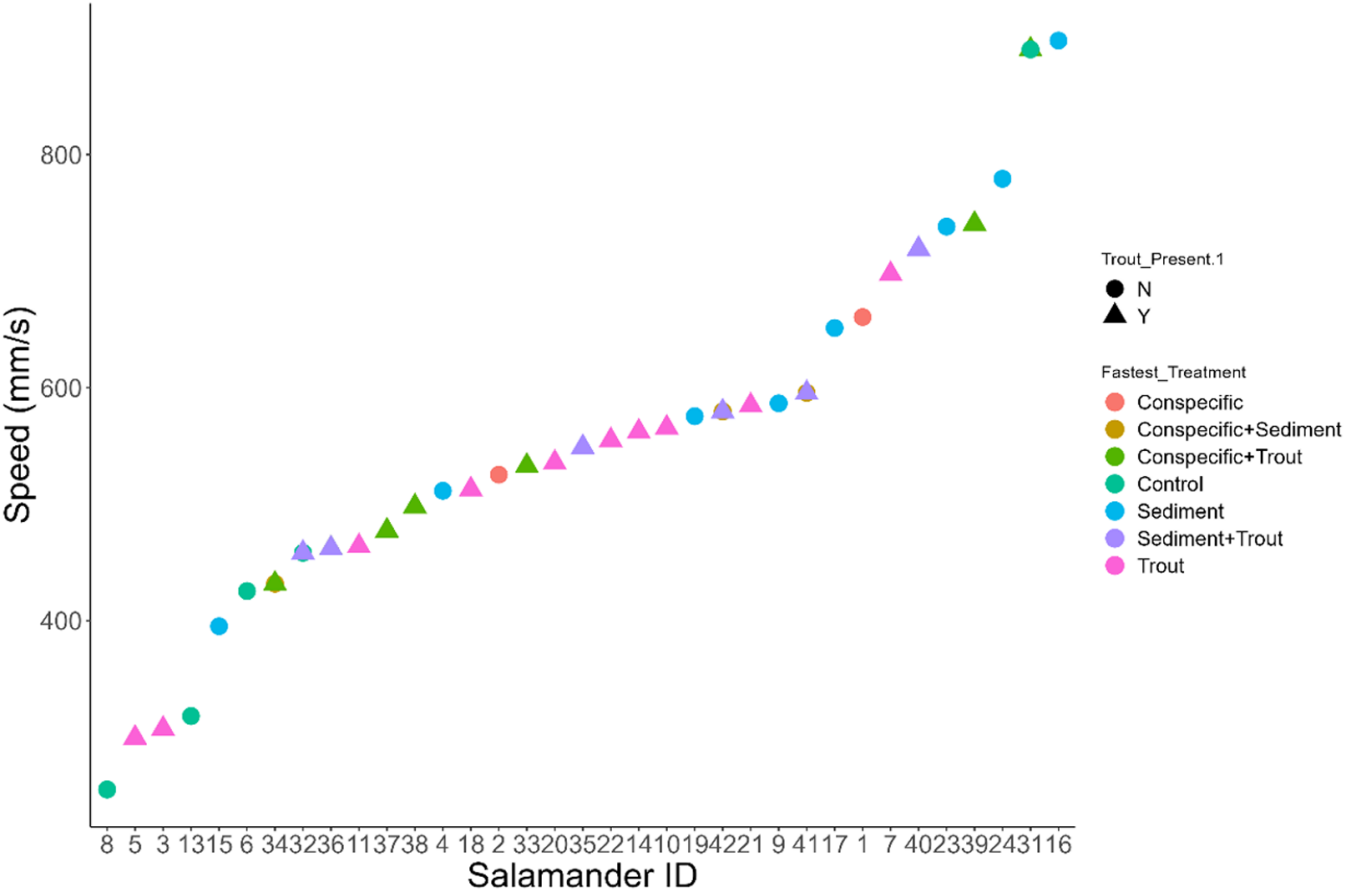
Th‐fastest speed of each individual *Dicamptodon tenebrosus* larva, with symbol shape and color denoting the treatment in which the fastest speed was recorded.

Treatment was a significant predictor of the movement speed of *Dicamptodon tenebrous* larvae (ANOVA *F*_(6,377)_ = 9.1, *p* < .0001). Larvae swam fastest in the combined presence of sediment and trout chemical cues (ST) with an escape rate nearly double that of individuals experiencing control conditions (400± 204 mm/s vs. 204 ± 123 mm/s, Tukey HSD *p* < .0001), or when no sediment, trout, or conspecific cues were present (Figure 1). Across multiple treatments, the presence of trout chemical cues also led to significantly higher escape speeds (366 ± 183 vs. 263 ± 155 mm/s, ANOVA *F*_(1,382)_ = 34.75, *p* < .0001, Figure 1). When looking at the top speed of individuals across all trials they performed, the majority (61.2%) occurred when trout cues were present (Figure 2).

A GLM including all larvae revealed that escape trials performed under the conspecific–trout treatment (*p* = .015, coefficient estimate = 90, SE = 34), sediment‐trout treatment (*p* < .0001, coefficient estimate = 150, SE = 35), and trout treatment (*p* = .004, coefficient estimate = 91, SE = 32) led to significant increases in movement speed. Other significant factors were water temperature (*p* = .009, coefficient estimate = 8, SE = 3), where cooler temperatures led to faster speed, and injury status (*p* < .0001, coefficient estimate = 84, SE = 23) where injured larvae were slower than uninjured larvae. A second GLM, excluding injured larvae (*n* = 5), found that cooler water temperature (*p* = .03, coefficient estimate = 7, SE = 4), the sediment–trout treatment (*p* < .0001, coefficient estimate = 156, SE = 39), and the trout treatment (*p* = .007, estimate = 97.0, SE = 36) led to significantly faster movement speed in uninjured larvae.

A GLMM with movement speed as the response variable, treatment as the fixed variable, and size, water temperature, and injury status as random variables showed treatment to be a significant predictor of movement speed: sediment treatment (*p* = .029, coefficient estimate = 67, SE = 31), sediment–trout treatment (*p* < .0001, coefficient estimate = 108, SE = 36), and trout treatment (*p* = .014, coefficient estimate = 77, SE = 31).

**FIGURE 3:**
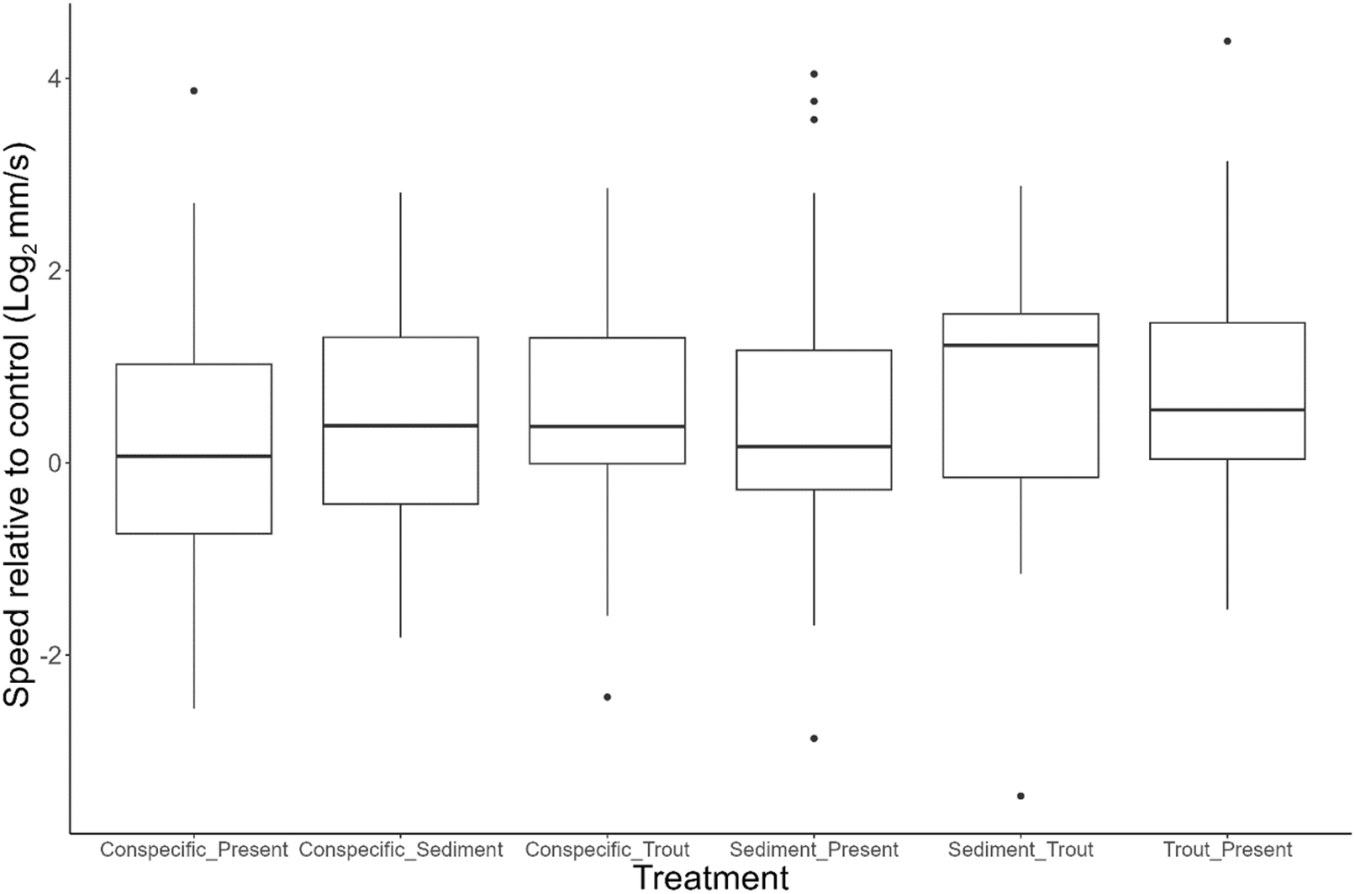
Speed changes across treatments in *Dicamptodon tenebrosus* larvae.

In addition to group‐level changes, individual performance varied across the different treatments. All noncontrol treatments led to an increase in individual performance compared to control conditions. Median percent speed increases ranging from 5% for conspecific treatments to 46.4% for the sediment‐trout cue treatment in comparison to the speed observed under control conditions (Figure 3). Median values are reported here due to the nonnormal distribution of treatment effects on speed compared to controls.

Within each treatment, different proportions of individuals increased or decreased speed compared to controls. The sediment–trout, conspecific–trout, and trout‐only treatments led to significantly more salamanders moving faster than they did under control conditions compared to the conspecific‐only, conspecific–sediment, and sediment‐only treatments (*χ* ^2^_(6df)_ = 9.8, *p* = .02). A GLM looking at the main and interactive effects of temperature classes and the different treatments showed that the sediment–trout treatment led to a significantly greater change in speed (117% increase) compared to controls than other treatments (*p* = .04), while the interactions between low temperatures and the sediment–trout treatment led to a slight, statistically significant decrease (2.16%) in speed compared to controls (*p* = .02).

**FIGURE 4:**
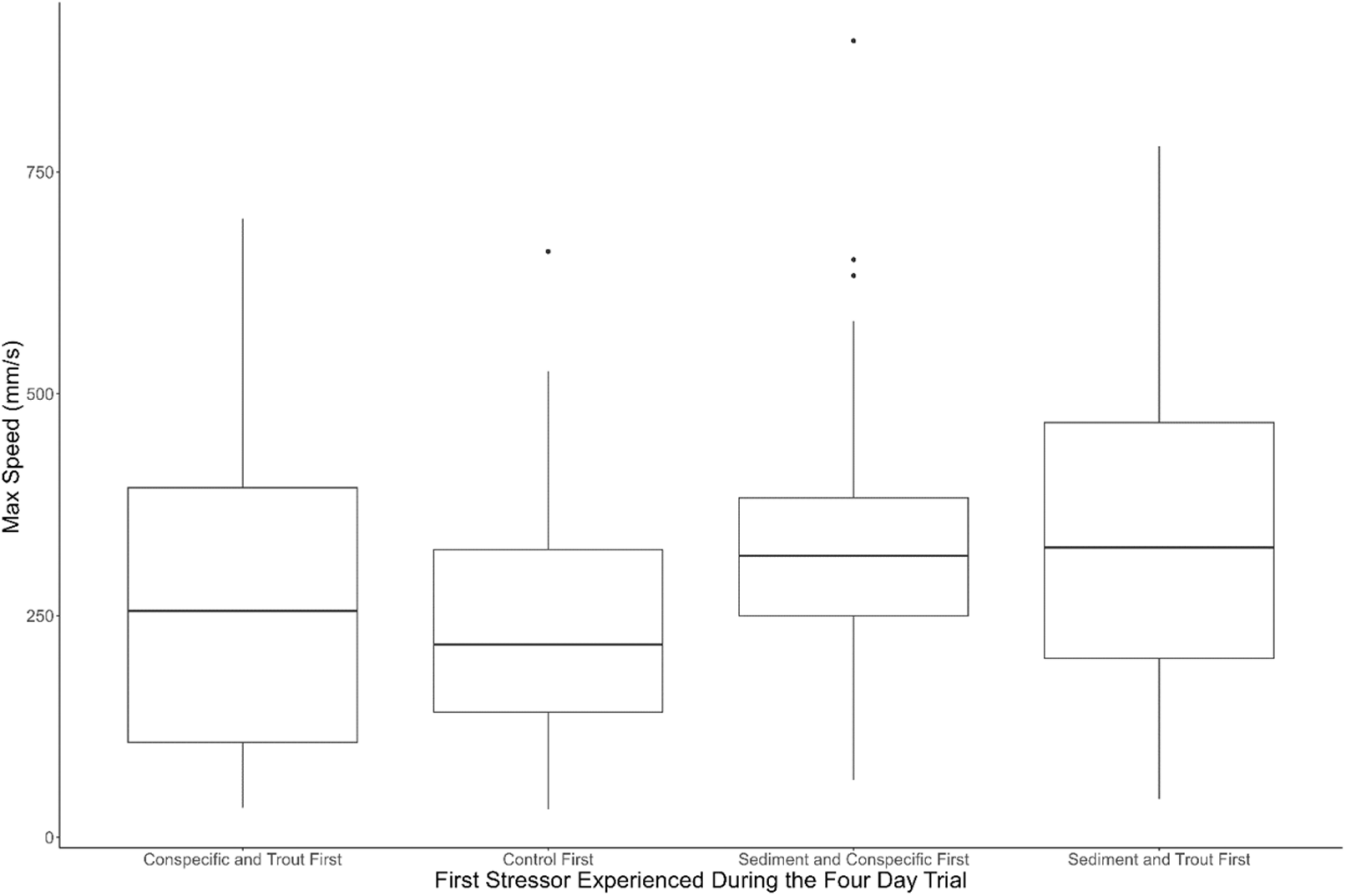
Differences in max speeds across four trials based on the first treatment experienced by larvae.

In addition to the treatments themselves, groups of salamanders experienced the stressors in different orders across the 4‐day escape assay trials given the study design. There was no effect of the order of stressors in the single‐stressor assay on movement speed, but there was a significant impact of the first stressor each group of salamanders experienced at the beginning of the 4‐day trials on the max speeds of the salamanders on the rest of the trials they underwent during the 4 day period (ANOVA *F*_(3,1178)_ = 23.54, *p* < .001, Figure 4).

**FIGURE 5:**
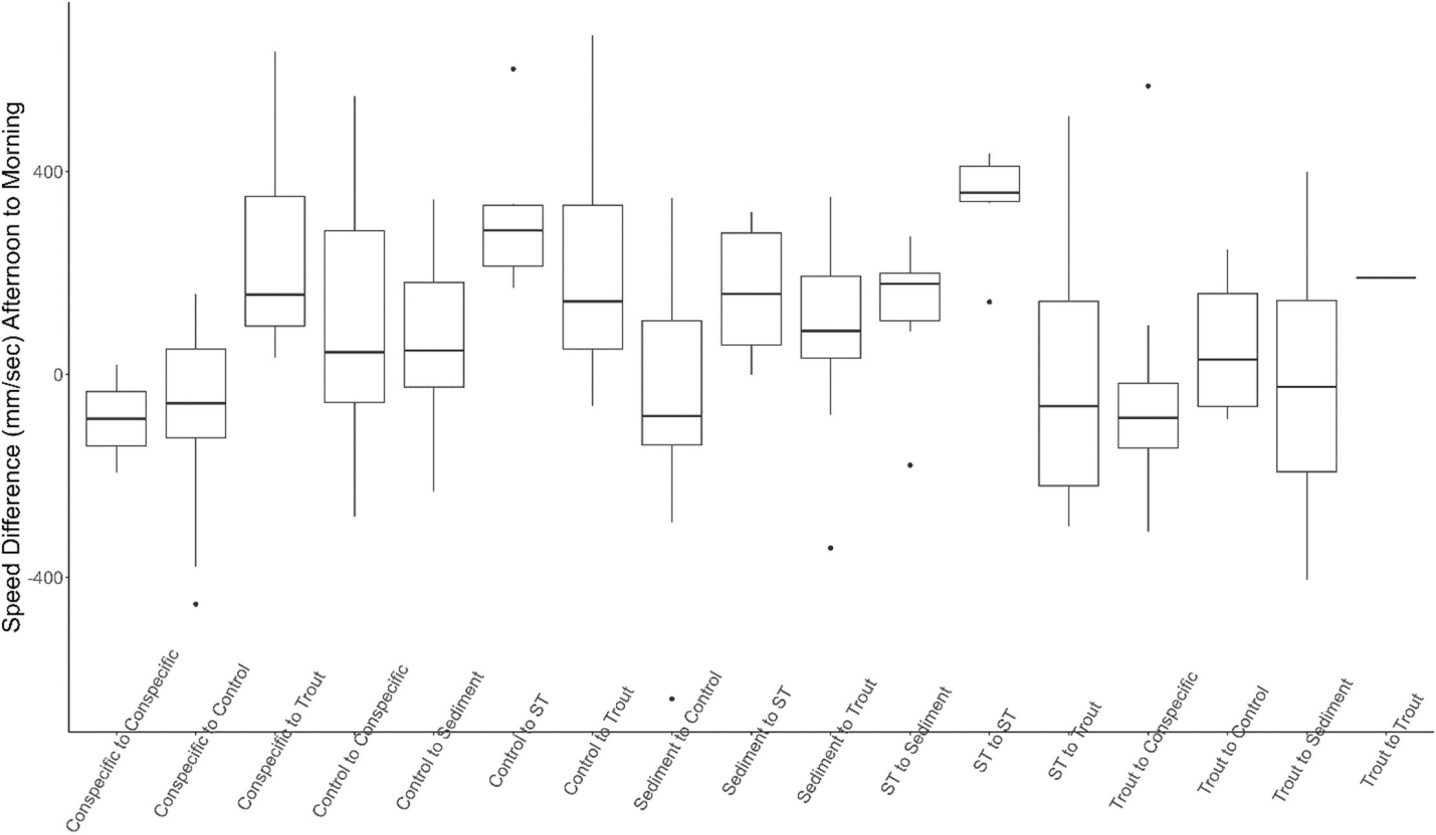
Speed changes between morning and afternoon trials for *Dicamptodon tenebrosus* larvae experiencing different stressor combinations. The first trial listed was conducted in the morning and the second trial listed was conducted in the afternoon.

When looking at responses of an individual on a given day, the combination and order of stressors had a significant effect on the speed of the individual on the second trial. Specifically, the combination of stressors (ANOVA *F*_(16,171)_ = 3.7, *p* < .001) and the identity of the first stressor experienced (ANOVA *F*_(4,136)_ = 3.1, *p* = .017, Figure 4) had a significant impact on the speed of the salamander on the second trial. Of all the potential morning‐to‐afternoon stressor combinations, exposure to sediment and trout (ST) at both times led to the greatest increase in speed on the second trial (~400 mm/s, Figure 5). Exposure to conspecific cues in the first trial led to significantly slower speeds during a subsequent trial regardless of the stressor present during that second trial than exposure to either sediment and trout conditions (*p* = .046, Tukey HSD, diff = 209.38) or control conditions (*p* = .044, Tukey HSD, diff = 202.11) during the first trial.

**FIGURE 6:**
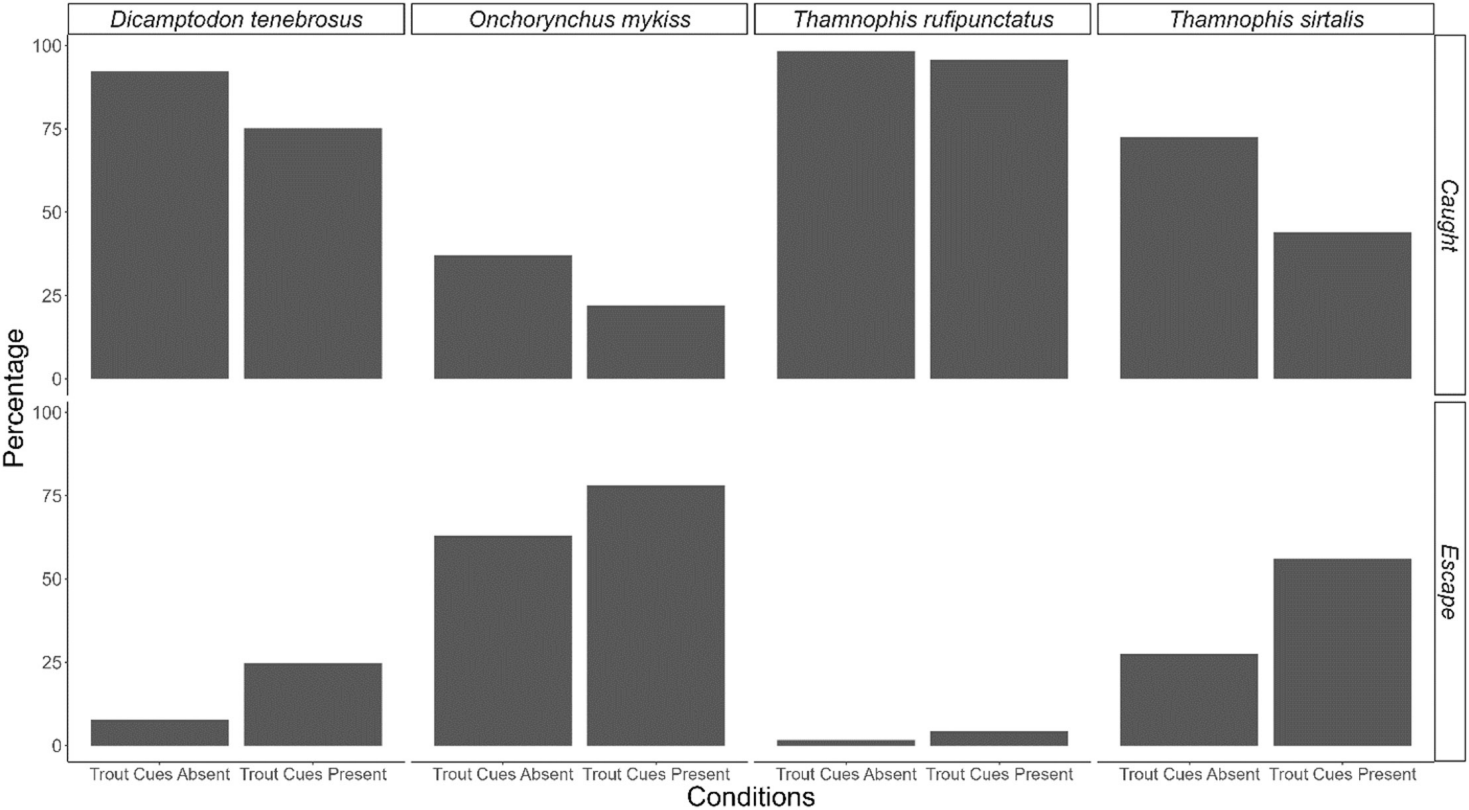
Predicted escape success by *Dicamptodon tenebrosus* larvae in the presence and absence of trout chemical cues from the aquatic garter snake (*Thamnophis rufipunctatus*), steelhead trout (*Oncorhynchus mykiss*), terrestrial garter snake (*Thamnophis sirtalis*), and conspecific *D. tenebrosus*.

In comparison to strike speeds from potential predators, significantly more larvae exposed to trout chemical cues would have escaped from an average‐speed trout strike (*χ* ^2^_(1df)_ = 29.3, *p* < .0001, Figure 6), conspecific strike (*χ* ^2^_(1df)_ = 19.3, *p* < .0001, Figure 6), and the terrestrial garter snake, *Thamnophis sirtalis*, strike (*χ* ^2^_(1df)_ = 9, *p* = .003, Figure 6). Here, it is worth noting that although the scenario where larvae would experience a trout strike without the presence of trout chemical cues appears unlikely, it could occur if trout are downstream of larvae prior to the strike attempt. The presence of visual cues from conspecifics, however, did not lead to a significant increase in escape success from an average‐speed conspecific strike, as the number of individuals that escaped successfully was not greater for treatments with conspecific cues than those without. In fact, more individuals in the absence of conspecific visual cues (*n* = 38), than in the presence of conspecific visual cues (*n* = 15), would have escaped an average‐speed conspecific strike. Few individuals were fast enough to escape an average strike from an aquatic garter snake, *Thamnophis rufipunctatus*, but those that were did so under the conspecific–trout treatment (*n* = 1), sediment–trout treatment (*n* = 5), sediment treatment (*n* = 3), and conspecific–sediment treatment (*n* = 1). When broken by presence and absence of trout chemical cues, six individuals in the presence of cues could have escaped an average‐speed strike by *T. rufipunctatus*, while four in the absence of cues would have escaped from an average‐speed strike from *T. rufipunctatus*.

### 3.2 HSP Expression

**FIGURE 7:**
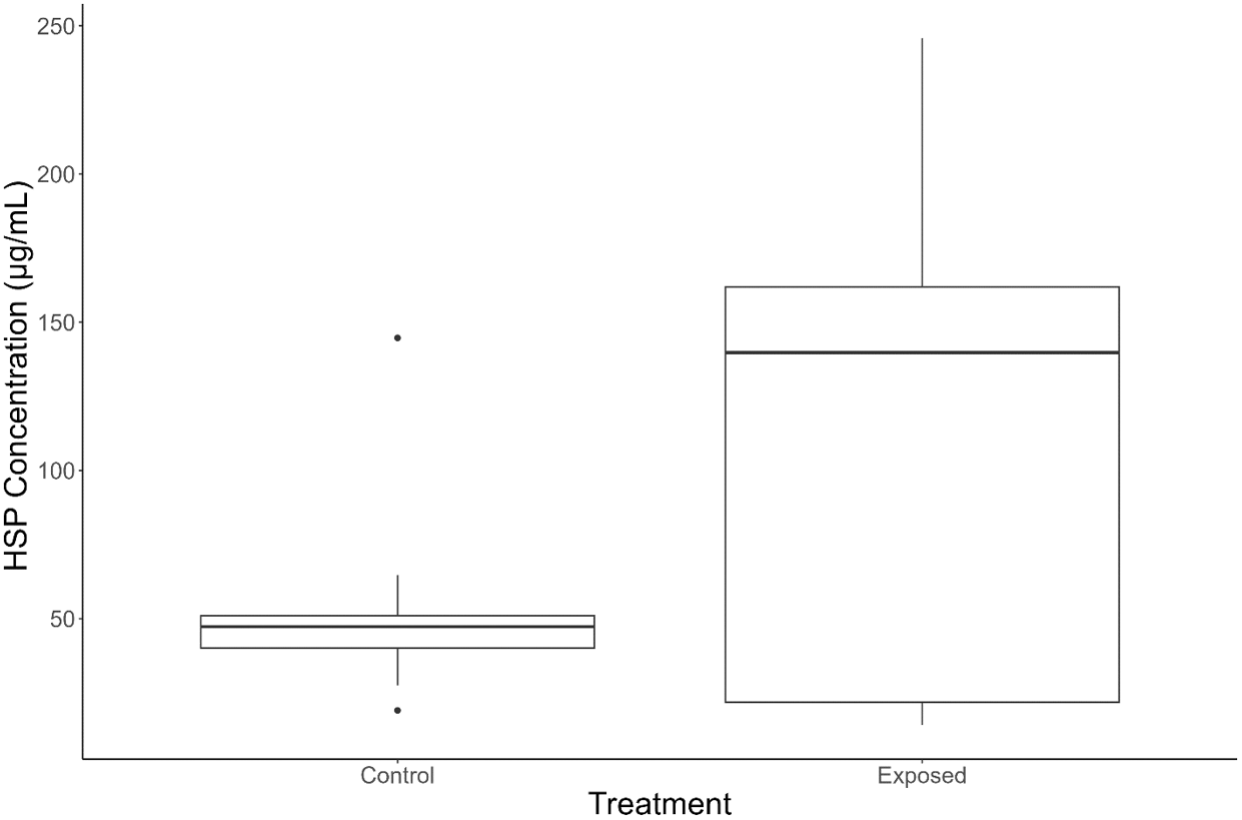
Heat‐shock protein (HSP70) levels of *Dicamptodon tenebrosus* larvae exposed under control or low‐flow exposure (elevated temperature) conditions.

Larvae in the exposed treatment group had higher mean and median HSP expression levels measured as HSP70 protein concentration (μg/mL) compared to controls although the difference between these two levels of expression was not statistically significant (*p* = .12, *F* value = 2.81, df = 1, ANOVA, Figure 7). There was significantly greater variation in expression between exposed and control individuals (*p* < .01, *F* value = 9.75, df = 1, Levene's test). Heat‐shock protein expression did not vary significantly between age classes (*p* = .64, ANOVA), or between injured and noninjured individuals (*p* = .47, ANOVA). There was no correlation between total length and HSP expression in control individuals or exposed individuals.

## 4 DISCUSSION

We observed how a biotic stressor, chemical cues from a potential predator, and an abiotic stressor, suspended sediment, increased movement speed in larvae of the giant Pacific salamander, *D. tenebrosus*, when present jointly and in isolation. We also observed that exposure to simulated summer, low‐flow conditions leads to greater variability in HSP expression in the same species of larvae. Both the presence of trout chemical cues and the addition of suspended sediment led to significant increases in the movement speed of *D. tenebrosus* larvae during escape maneuvers in comparison to controls. The presence of both stressors in combination led to even higher speeds than either stressor in isolation, although the difference between speeds observed under these treatments was insignificant (Figure 1). This pattern suggests that the two stressors have additive but not synergistic or antagonistic (Crain et al., 2008; Gunderson et al., 2016) influences on salamander behavior, and that the combined presence of trout chemical cues and suspended sediment pose more threatening circumstances for *D. tenebrosus* larvae.

Amphibian larvae respond behaviorally and physiologically to chemical cues from potential predators (Katzenberger et al., 2014; Kiesecker et al., 1996; Petranka et al., 1987). For example, when predatory chemical cues are present, amphibian larvae of multiple species avoid cue areas, decrease unnecessary movement, and/or increase burst movement speed (Ferrari et al., 2008; Katzenberger et al., 2014; Kiesecker et al., 1996; Petranka et al., 1987). Amphibian larvae appear capable of distinguishing between different predatory chemical cues and incorporating environmental variables, including time of day (Middlemis Maher et al., 2013) and presence of injured conspecific cues (Petranka et al., 1987), into their risk assessment of potential predation. Additionally, predatory cues have been shown to elicit morphological and physiological changes in amphibian larvae after sufficient exposure to the cue. Specific changes include altered tail shape, color, and size (Narayan et al., 2013; Wilson et al., 2005), cortisol‐based “fear” responses (Narayan et al., 2013), and increase in oxidative stress levels (Janssens & Stoks, 2013; Pinya et al., 2016). These results show how some amphibians may respond to the presence of predators by changing to better avoid predation (Narayan et al., 2013; Wilson et al., 2005), although the response can be physiologically detrimental (Pinya et al., 2016).

With respect to the species of this study, *D. tenebrosus*, previous work has shown that larvae increase cover‐seeking behavior in response to trout chemical cues (Rundio & Olson, 2003). We did not observe any difference in cover‐seeking behavior between treatments, but that may be due to differences in study design. In Rundio and Olson (2003), trout cues were coming from upstream representing a more distant and potentially approaching threat, whereas in our setup, trout were exposed to chemical cues in a smaller area. In our experiment, the presence of the cue would more closely simulate a trout that was in the immediate vicinity of the salamander with no clear direction to where it was at that moment. Furthermore, in our design, the cue was paired with a physical disturbance leading to the measured burst movement speed of the larvae. This disturbance was absent in the experiment performed by Rundio and Olson (2003).

In addition to responding to chemical cues from predators, aquatic amphibians are susceptible to increasing loads of suspended sediment. In a large‐scale “natural experiment” in streams across northern California, Welsh and Ollivier (1998) documented the populations of multiple stream‐residing amphibians before and after a 1989 storm event that significantly increased loads of suspended fine sediment in stream ecosystems. The species studied included larvae of tailed frog (*Ascaphus truei*), *D. tenebrosus* larvae, and paedeormorphs, and larvae and adults of southern torrent salamander (*Rhyacotriton variegatus*). Each species responded to sediment differently, but all displayed population decreases following the storm event (Welsh & Ollivier, 1998).

Sediment is particularly challenging for larval amphibians that use small gaps in substrate as refuge, as the sediment fills in the areas they require for protection from predation (Welsh & Ollivier, 1998; Wood & Armitage, 1997). Additionally, suspended sediment can be ingested by filter feeding larvae and inhibit food ingestion (Wood & Richardson, 2009). Wood and Richardson (2009) observed slower growth and reduced survival following sediment ingestion in western toad (*Bufo boreas*) tadpoles. Furthermore, when larvae rely primarily on gills for respiration, as is the case for *D. tenebrosus* and many other amphibians, suspended sediment can lead to heightened metabolic rates suggestive of less efficient gas exchange (Unger et al., 2021).

The effects of suspended sediment on respiration are better studied in fish and aquatic invertebrates (Harvey et al., 2009; Kemp et al., 2011; Rosewarne et al., 2014). Sutherland and Meyer (2007) showed that two species of highland minnows, *Cyprinella galactura* and *Erimonax monachu*, displayed moderate and high levels of gill tissue damage when exposed to levels of suspended sediment greater than 100 mg/mL for 21 days. In salmonids, increasing sediment has also been associated with gill tissue damage and increasing coughing frequency, a sign of sublethal respiration issues (Bash et al., 2001). Other documented effects of sediment on aquatic organisms include decreased growth (Harvey et al., 2009), changes in blood physiology (Bash et al., 2001), and changes in behavior (Harvey et al., 2009; Kemp et al., 2011). One such behavioral change is avoidance of high sediment areas (Harvey et al., 2009). This avoidance suggests that aquatic organisms recognize the threat posed by sediment in the environment and are seeking a way out of the high sediment area. This readiness to escape a high sediment area can help explain the greater movement speed of *D. tenebrosus* larvae under high sediment conditions (Figures 1, 2, 3). We also noted that larvae left the water and climbed up on the cover objects in the enclosure during trials only twice, both of which occurred during a sediment trial. Similarly, “wandering” behavior, that is, larvae moving around the enclosure of their own volition before any disturbance occurred, only happened during sediment trials. These unusual behaviors further support the idea that the larvae are uncomfortable in the sediment and seeking a way to escape.

This concept of sediment avoidance is also supported by the pattern of increasing movement speed of *D. tenebrosus* larvae in the presence of both sediment and trout chemical cues (Figures 1, 2, 3). Although speed when both stressors were present was not significantly higher than either stressor in isolation, the pattern suggests that the stressors have an additive effect and jointly are pushing salamander to higher speeds to escape the dual threat of predation and damage from suspended sediment. Additionally, it appears that the order in which the larvae experience the stressors may influence their response, especially when incorporating different co‐occurring stressors. Specifically, we observed a significant difference in movement speed between groups in the multistressor assay across treatment types. Interestingly, two of the four groups that moved the fastest during the duration of the trial were exposed to sediment in their first trial, while the group with the slowest speeds was exposed to control conditions during the first trial. The order in which organisms experience stressors can have an impact on how they respond (Crain et al., 2008; Todgham et al., 2005), and it is possible that the sediment had a priming effect on the salamanders that made them more responsive to stressors over the next few days. Such possible priming is further supported by how exposure of larvae to sediment and trout chemical cues in both the morning and afternoon of a given day led to the highest observed increases in speed across the two trials (Figure 5). This effect could reflect the detrimental effects of sediment discussed previously and the avoidance of high sediment areas by *Dicamptodon tenebrosus* observed by Harvey et al. (2009). Future studies should investigate the pathways through which larval *D. tenebrosus* is responding to each of these stressors to better understand the mechanisms leading to the heightened escape response we observed. For example, it would be informative to see if these stressors are impacting important physiological metrics such as metabolic rate (Burton et al., 2011) and oxidative stress levels (Speakman et al., 2015). Both metrics are responsive to changes in the environment (Beaulieu & Costantini, 2014; Burton et al., 2011), and any disruptions of their usual levels can have significant fitness consequences for impacted organisms (Angilletta & Sears, 2000; Ardia et al., 2012).

In addition to sediment and chemical cues from trout, we also looked at the impacts of visual predatory cues from conspecifics and natural temperature changes by performing trials in the morning and afternoon. We did not, however, find any significant impact of the presence of conspecifics on the escape behavior of *D. tenebrosus* larvae in the trial when the cues were presented. We did observe, however, that individuals exposed to conspecific visual cues on the first of two trials in a day moved slower in the second trial compared to salamanders exposed to control conditions and those exposed to the combined presence of sediment and trout chemical cues. Temperature had a significant but small impact on movement speed, with a 1°C increase in temperature corresponding with roughly a 7 mm/s decrease in movement speed. We also checked to see if there was any difference between morning and afternoon trials that could be attributed to variations in diurnal pattern (Middlemis Maher et al., 2013) rather than temperature but found that no significant differences between times of day were observed.

The lack of a response to conspecifics would suggest either the larvae are not detecting the conspecific through the glass, or they are detecting the conspecific, but do not consider it a threat at the time. The former would seem unlikely given most aquatic salamander larvae such as *D. tenebrosus* have decent vision and use it to detect and differentiate between prey in conjunction with other sensory systems such as chemoreceptors or lateral line systems in aquatic environments (Roth, 2012). Vision also appears to dominate, at least in prey capture, for many species in environments with sufficient light and water clarity (Roth, 2012). This would suggest that in trials where only the visual cue from conspecifics was present, the other salamander would be able to see and respond to the potential predator (Melotto et al., 2021). Consequently, the latter possibility that the salamanders did not perceive the conspecific as a threat at the time seems more likely, especially given the hunting strategies of this species. *D. tenebrosus* is a primarily nocturnal hunter (Parker, 1994), so it is possible that larvae felt less threatened by conspecifics during the morning and afternoon hours because it would be unusual for a conspecific to actively pursue them at that time (Middlemis Maher et al., 2013). Alternatively, larvae may have been able to recognize the barrier between them and the conspecific. On multiple occasions, larvae came to rest after a disturbance directly next to the conspecific in the glass container, solely separated by the glass wall of the container. Such proximity suggests larvae were aware of the physical barrier to the other salamander, although this behavior could also reflect the prior hypothesis.

The potential delayed effect of conspecific cue exposure on the speed of subsequent trials also fits with the hypotheses described above. If salamanders recognize conspecifics in morning trials, but do not perceive a significant threat at that time, it is still possible they are more wary in the second trial of the day leading to the decreased movement speed we observed. That is, with *Dicamptodon* hunting time approaching during the afternoon trials, it is possible that larvae exposed to conspecifics in the morning in the enclosure associated the area with the possibility of predation that was now a more likely possibility (Middlemis Maher et al., 2013; Parker, 1994).

In addition to comparing speeds under different treatments, we also compared speed to known average strike speeds of known predators of *D. tenebrosus* including steelhead trout (Parker, 1993; Webb & Zhang, 2011), the terrestrial garter snake (*Thamnophis sirtalis*) (Alfaro, 2002; Nussbaum & Clothier, 1973), aquatic garter snake (*Thamnophis rufipunctatus*) (Alfaro, 2002; Nussbaum & Clothier, 1973), and conspecifics (Kleinteich et al., 2014; Munshaw et al., 2014; Reilly & Lauder, 1992). In line with our previous finding that trout chemical cue increases movement speed in *D. tenebrosus* larvae, we also saw that more larvae would move fast enough to evade a trout strike when chemical cue is present compared to when it is absent (Figure 6). This suggests that whatever pathway leads to increased speed in larvae when trout are nearby could be partially tailored to the predatory threat. Other amphibians have displayed predator‐specific responses and behaviors based on the nature of the threat (Brodie, 1977; Ducey & Brodie, 1983; Marchisin & Anderson, 1978). Consequently, it is reasonable to assume that *D. tenebrosus* larvae also display different escape behaviors given the predator in question and circumstances at hand. Such adaptations for different predators, however, do not negate the capacity for a behavior to help with multiple predators, especially when the behavior is as general as a burst speed escape (Cooper & Blumstein, 2015; Katzenberger et al., 2014; Melotto et al., 2021). In this experiment, this capacity is observable in the significantly higher number of larvae exposed to trout chemical cues also moving quickly enough to evade strikes from conspecifics and *T. sirtalis* (Figure 6). The strike speed of *T. rufipunctatus*, however, was faster than most of the speeds we recorded, begging the question of whether *D. tenebrosus* has another level of speed that it employs if it detects presence of *T. rufipunctatus*, or if it employs a different antipredator strategy (Brodie, 1977; Ducey & Brodie, 1983; Marchisin & Anderson, 1978). Future studies should incorporate predatory cues, both visual and chemical, from a wider range of predators to see if *D. tenebrosus* alters its movement speed and behavioral patterns in the face of different threats.

The differences in heat‐shock protein expression levels we observed between exposed and control groups, although not significant, suggest that HSPs may play a role in *D. tenebrosus* larvae responses to challenging summer conditions (Figure 7). Additionally, one important factor in physiological responses is the duration of stressor (Crain et al., 2008; Gunderson et al., 2016), so it is possible that a longer exposure for the exposed group would produce more divergent results. Furthermore, we observed greater variance in the exposed group, highlighting physiological diversity at the inter‐individual level in the face of challenging conditions (Spicer & Gaston, 1999) in *D. tenebrosus* that could influence which individuals survive the summer months (Nussbaum & Clothier, 1973).

The fact that HSPs were not found in tissue samples of some individuals likely reflects the possibility that HSPs were not expressed in the specific tissues of every tail clip. One of the drawbacks of using less invasive techniques such as minimally invasive tail clips is the possibility that certain markers will be absent from subsequent tests of tissue samples (Milanovich & Maerz, 2012). Nevertheless, the use of tail clips allows for repeated measurements of physiological markers from populations and can allow future studies to document HSP levels in *D. tenebrosus* over longer time scales with larger sample sizes. Additionally, future studies could incorporate HSP analyses following different stressor exposures (e.g., increased sediment) to get a more complete picture of the importance of HSPs for *D. tenebrosus* in the face of current and future environmental stressors. It will be important to know whether the levels of HSPs produced under worsening conditions due to climate change (IPCC, 2021) are sufficient to counter the damage caused by environmental stress (Kültz, 2005; Todgham et al., 2005).

## 5 CONCLUSION

In summary, we performed a nonlethal, filed‐based assay of an ecologically important trait under multiple‐stressor conditions by measuring movement speed of *D. tenebrosus* larva in the presence of biotic and abiotic stressors. We observed that *D. tenebrosus* larvae can detect and respond to chemical cues from steelhead trout with increased burst escape speed following a disturbance and that a similar increase in movement speed occurs when *D. tenebrosus* experiences increased suspended sediment. Additionally, a further increase in speed occurs when these two stressors occur in tandem, suggesting they are additive in nature and may act on similar pathways to influence *D. tenebrosus* movement. The increase in speed associated with the presence of trout chemical cues is sufficient to significantly increase the chance a *D. tenebrosus* larvae will escape a predatory strike from a steelhead trout, terrestrial garter snake, or conspecific. Interestingly, visual presence from a potential conspecific predator did not lead to a similar change in escape speed and behavior, possibly because the larvae did not perceive the conspecific as a threat in the specific conditions posed by our trials.

Larvae were only slightly impacted by changes in water temperature within the natural range experienced by the creek currently, with warmer temperatures leading to marginally lower speeds. With summer temperatures increasing in Fox Creek (IPCC, 2021), however, it is possible that future high temperatures may hinder larval escape capabilities. These increasing temperatures are also likely to increase the HSP response we observed in individuals in exposed and in‐stream treatments. Those who were exposed had more variable levels of HSP expression that tended to be higher than controls. With continued warming and altered precipitation patterns (Allen et al., 2011; IPCC, 2021; Reid & Dunne, 1984), it is possible that these HSP systems will be pressed to their limits.

Given the multifaceted nature of stressors in aquatic environments (Crain et al., 2008; Gunderson et al., 2016), it is important to document the interactive effects of simultaneous stressors in controlled lab settings and more dynamic field environments. These types of studies help bridge the studies of ecology and physiology and are thereby important tools for conservation efforts (Cooke et al., 2013; Madliger et al., 2018; Wikelski & Cooke, 2006).

## AUTHOR CONTRIBUTIONS

**Oliver Coyle:** Conceptualization (lead); data curation (lead); formal analysis (lead); funding acquisition (lead); investigation (lead); methodology (lead); project administration (lead); resources (lead); software (lead); supervision (lead); validation (lead); visualization (lead); writing – original draft (lead); writing – review and editing (lead). **Vance T. Vredenburg:** Conceptualization (supporting); funding acquisition (supporting); investigation (supporting); methodology (supporting); resources (supporting); supervision (supporting); writing – review and editing (supporting). **Jonathon H. Stillman:** Conceptualization (supporting); data curation (supporting); formal analysis (supporting); funding acquisition (supporting); investigation (supporting); methodology (supporting); project administration (supporting); resources (supporting); supervision (supporting); writing – review and editing (supporting).

## FUNDING INFORMATION

Many thanks to the Genentech Foundation and ARCS Foundation for financial support.

## CONFLICT OF INTEREST STATEMENT

The authors report no conflict of interest in this research.

## LENGTH JUSTIFICATION

This study looked at two distinct components of larval *D. tenebrosus* responses to environmental stressors which required separate methodologies and analysis in addition to a lengthier discussion which necessitated the greater length of this document.

## Data Availability

Data will be accessible from the Dryad repository: http://doi.org/10.5061/dryad.c59zw3rff.
